# Supplementation of eicosapentaenoic acid-rich fish oil attenuates muscle stiffness after eccentric contractions of human elbow flexors

**DOI:** 10.1186/s12970-019-0283-x

**Published:** 2019-04-15

**Authors:** Yosuke Tsuchiya, Kenichi Yanagimoto, Hisashi Ueda, Eisuke Ochi

**Affiliations:** 1 Faculty of Modern life, Teikyo Heisei University, Tokyo, Japan; 2 Food Function R&D Center, Nippon Suisan Kaisha, Ltd., Tokyo, Japan; 3 Faculty of Health and Medical Science, Teikyo Heisei University, Chiba, Japan; 4 Faculty of Bioscience and Applied Chemistry, Hosei University, 3-7-2, Kajino, Koganei, Tokyo, Japan

**Keywords:** Eicosapentaenoic acid, Omega-3 polyunsaturated fatty acids, Shear wave elastography, Shear elastic modulus, Muscle function, Muscle swelling, Muscle soreness, Echo intensity

## Abstract

**Background:**

This study aimed to investigate the effect of supplementation of fish oil rich in eicosapentaenoic acid (EPA) and docosahexaenoic acid (DHA) on the damage of the biceps brachii after eccentric contractions (ECCs) of the elbow flexors, particularly focusing on muscle stiffness.

**Methods:**

Sixteen men were included in this double-blind, placebo-controlled, parallel design study and the participants were randomly assigned to the EPA and DHA supplement group (EPA, *n* = 8) and placebo group (PL, *n* = 8). They consumed either EPA 600 mg and DHA 260 mg per day or placebo supplement for 8 weeks prior to exercise. Moreover, they performed six sets of 10 ECCs at 100% maximal voluntary contraction (MVC) using a dumbbell. Changes in MVC torque, range of motion (ROM), upper arm circumference, muscle soreness, muscle echo intensity, and muscle stiffness were assessed before exercise; immediately after exercise; and 1, 2, and 5 days after exercise.

**Results:**

MVC torque and ROM were significantly higher in the EPA group than in the PL group after ECCs (*p* < 0.05). Muscle soreness, upper arm circumference, and muscle echo intensity were significantly higher in the PL group than in the EPA group after ECCs (*p* < 0.05). In addition, muscle stiffness at 150° was significantly higher in the PL group than in the EPA group immediately after ECCs (*p* < 0.05).

**Conclusion:**

The present study showed that EPA and DHA supplementation has a positive role in inhibiting muscle stiffness after ECCs.

**Trial registration:**

This trial (UMIN000028165) was registered on 10th/July/2017.

## Background

Fish oil contains omega-3 polyunsaturated fatty acids, specifically, eicosapentaenoic acid (EPA) and docosahexaenoic acid (DHA). These fatty acids are a major component of the cell membrane and accumulate in the muscle cellular membrane after intake of fish oil [1]. Although the mechanism is unclear, EPA and DHA supplementation from fish oil may inhibit muscle damage after exercise by protecting the muscle cell membrane and promoting an anti-inflammatory response. Previous studies have shown that EPA and DHA supplementation inhibited muscle damage after eccentric contractions (ECCs) [2–5]. A recent study also confirmed that the intake of EPA and DHA inhibited the loss of muscle strength, limited range of motion (ROM), development of DOMS, and increase in serum interleukin (IL)-6 levels after 30 maximal ECCs of elbow flexors [5].

Recent studies have revealed that muscle stiffness increases after transient ECCs [6, 7]. Elastographic techniques can be used to assess the shear elastic modulus (i.e., elasticity) of a localized area in the muscle at rest [8–10] and during isometric contraction [9, 11] and passive stretching [12, 13]. Lacourpaille et al. [6] showed that muscle stiffness of the biceps brachii and brachialis increased at 1 h after ECCs using elastographic techniques. The increase in muscle stiffness after ECCs is associated with the release of Ca^2+^ owing to cell membrane damage along with sarcomere disruption during ECCs [6]. However, no study has reported about the effect of EPA and DHA supplementation on muscle stiffness after ECCs.

Thus, the present study aimed to investigate the effect of EPA and DHA supplementation on muscle stiffness, particularly in the biceps brachii, after ECCs of the elbow flexors. Muscle function, ROM, muscle soreness, upper arm circumference, and muscle echo intensity after ECCs were also assessed. Moreover, we hypothesized that EPA and DHA supplementation inhibits the increase in muscle stiffness after ECCs, decrease in muscle strength, limited ROM, development of muscle soreness, and increase in upper arm circumference and muscle echo intensity.

## Methods

### Subjects

A total of 16 healthy men were recruited for this study. The participants were not allergic to fish and had no resistance training. Thus, they were requested not to participate in other clinical trials and interventions, such as massage, stretching, strenuous exercise, excessive consumption of food or alcohol, and intake of supplementations or medications during the experimental period. All participants were provided with detailed explanations of the study protocol prior to participation, and an informed consent was obtained. The present study was performed in accordance with the Declaration of Helsinki and was approved by the ethics committee for human experiments of Hosei University (ID: 2017–002). Moreover, it has been registered at the University Hospital Medical Information Network Clinical Trials Registry (UMIN-CTR identifier: UMIN000028165).

### Study design

The study used the double-blind, placebo-controlled, parallel-group trial design. Since this study design was similar to our previous studies [2, 5] in order to extend these previous studies, we conducted similar type of subjects and ECCs. The participants were randomly assigned to two groups using a table of random numbers to minimize the intergroup differences in terms of age, and body mass index (BMI). According to the previous studies [2, 5], the placebo (PL) group consumed daily placebo capsules for 8 weeks prior to an exercise experiment and for 5 days after the exercise, whereas the EPA group consumed EPA supplement capsules. The participants consumed the capsules for 62 days (including during exercise days). The sequence allocation concealment and blinding of participants and researchers were maintained throughout this period. Medication adherence was assessed using the daily record of the patients and via pill count at the end of the study. On the day of exercise testing, the markers of muscle damage were assessed using the nondominant arm before exercise. Immediately after these baseline measurements, the participants performed ECCs using the same arm. All measurements were performed immediately before exercise, immediately after exercise, and 1, 2, and 5 days after exercise. In addition, the nutrition status of all participants was assessed prior to supplement consumption and after the experimental testing on food frequency using a questionnaire based on food groups (FFQg version 3.5, Kenpakusha, Tokyo, Japan). In addition, serum fatty acid levels were measured, including EPA, DHA, arachidonic acid (AA), and dihomo-gamma-linolenic acid (DGLA) levels.

### Supplements

Based on previous studies [2, 3, 5] and considering the safety factor [14], the EPA group consumed eight 300-mg EPA-rich fish oil softgel capsules (Nippon Suisan Kaisha Ltd., Tokyo, Japan) per day, and the total consumption was 2400 mg per day (600-mg EPA and 260-mg DHA). The PL group consumed eight 300-mg corn oil softgel capsules per day (without EPA and DHA), and the total consumption was 2400 mg. The participants took the capsules within 30 min after each meal.

### Blood sample

The participants fasted for 8 h before a trained doctor obtained blood samples from the forearm [5]. The blood samples were allowed to clot at room temperature (25 °C) and were then centrifuged at 3000 rpm for 10 min at 4 °C. The serum was extracted and stored at − 20 °C until analysis. The serum levels of DGLA, AA, EPA, and DHA were measured.

### ECCs

For the ECCs, the participant sat on a preacher curl bench with his shoulder joint angle at 45° flexion. For the use of the dumbbell, the value of maximal voluntary contraction (MVC) measurement at 90° was converted to kg. The exercise consisted of six sets of 10 maximal voluntary ECCs of the elbow flexors with a rest period of 90 s between each set, as described in our previous study [2]. The dumbbell was handed to the participant at the elbow flexed position (90°), and the participant was instructed to lower it to a fully extended position (0°) at an approximately constant speed (30°/s) in time (3 s) with a metronome. The investigator then removed the dumbbell, and the participant returned his arm without the dumbbell to the start the position for the next ECC.

### MVC torque

For the measurement of MVC torque, the participant performed three 3-s MVCs at 90°, 110°, 130° of elbow joint angle with a 15-s rest during contractions. The peak torque of each angle contractions was used as the MVC torque. The torque signal was amplified using a strain amplifier (LUR-A-100NSA1; Kyowa Electronic Instruments, Tokyo, Japan). The analog torque signal was converted to digital signals with a 16-bit analog-to-digital converter (Power-Lab 16SP; AD Instruments, Bella Vista, Australia). The sampling frequency was set at 10 kHz. The measurement was based on a previous study [15].

### ROM of the elbow joint

To examine the ROM of the elbow joint, two elbow joint angles (extended and flexed) were measured using a goniometer (Takase Medical, Tokyo, Japan). The extended joint angle was recorded while the participant attempted to fully extend the joint with the elbow held by his side and the hand in supination [5, 16, 17]. The flexed joint angle was identified while the participant attempted to fully flex the joint from an equally fully extended position with the hand supinated. The ROM was calculated by subtracting the flexed joint angle from the extended joint angle.

### Muscle soreness

Muscle soreness in the elbow flexors was assessed using a 10-cm visual analog scale in which 0 indicated “no pain” and 10 “the worst pain imaginable” [5, 16, 17]. The participant relaxed his arm in a natural position. The investigator then palpated the upper arm using a thumb, and the participant indicated his pain level using the visual analogue scale. All tests were conducted by the same investigator who had been trained to use the same pressure over time between participants.

### Upper arm circumference

Upper arm circumference was assessed at 9 cm above the elbow joint using a tape measure while the participants were standing with the arms relaxed by their side [2]. The measurement marks were maintained during the experimental period using a semipermanent ink marker, and a well-trained investigator obtained the measurements. The average value of the three measurements was used for further analysis.

### Muscle echo intensity

For the measurement of muscle echo intensity, the elbow joint angles of the participants were set at 70°, 110°, and 150°. The B-mode ultrasound pictures of the upper arm were obtained using the biceps brachii via an ultrasound (Aixplorer version 4.2, Supersonic Imagine, France), and the probe was placed 9 cm from the elbow joint at the position marked for the measurement of the upper arm circumference. The same gains and contrast were used over the experimental period. The transverse images were transferred to a computer as bitmap (.bmp) files and analyzed using a computer. The average muscle echo intensity of the region of interest (20 × 20 mm) was calculated using the computer image analysis software that provided a gray scale histogram (0, black; 100, white) for the region, as described in a previous study [2].

### Muscle stiffness

Using the ultrasound shear wave elastography, muscle stiffness at 70°, 110°, and 150° elbow angle were measured, as previously described [6]. An ultrasonic scanner (Aixplorer version 4.2, Supersonic Imagine, France) in shear wave elastography mode with musculoskeletal preset was used. An electronic linear array probe (SL15–4, Supersonic Imagine) coated with water-soluble transmission gel was placed longitudinally on each muscle head. Muscle shear modulus (μ), a measure of normalized muscle stiffness, was calculated using the following equation: μ = ρVs2, where ρ is the muscle density (assumed to be 1000 kg/m^3^) and Vs is the velocity of shear wave propagation caused by focused ultrasound beam from the scanner. A 10-mm square map of the muscle shear modulus with a spatial resolution of 1× 1 mm was obtained with each ultrasound image. A representative value of the shear modulus for each muscle head was then identified via spatial averaging over a 5-mm diameter circle [18].

### Statistical analyses

All analyses were performed using the SPSS software version 22.0 (IBM Corp., Armonk, NY). Values were expressed as means ± standard deviation. MVC torque, ROM, muscle soreness, upper arm circumference, muscle echo intensity, and muscle stiffness of values on the time immediately after exercise, the days 1, 2, and 5 post-exercise were calculated based on relative changes from baseline. MVC, ROM, muscle soreness, upper arm circumference, muscle echo intensity, and muscle stiffness of the PL and EPA groups were compared using two-way repeated-measure analysis of variance. When a significant primary effect or interaction was found, Bonferroni’s correction was performed for post-hoc testing. The partial eta squared (η2) were calculated to demonstrate the effect size. A *p*-value of < 0.05 was considered statistically significant.

## Results

No significant differences were observed between the EPA and PL groups in terms of age, weight, and BMI (PL group, *n* = 8; age, 20.9 ± 0.4 years; height, 173.0 ± 6.0 cm; weight, 64.9 ± 6.8 kg; body fat, 15.9 ± 3.2%; and BMI, 21.7 ± 2.0 kg/m^2^; EPA group, n = 8; age, 21.9 ± 1.4 years; height, 174.9 ± 3.9 cm; weight, 67.1 ± 8.0 kg; body fat, 15.9 ± 4.6%; and BMI, 22.0 ± 3.0 kg/m^2^). Based on the results of the food frequency survey, no difference was observed between the PL group (energy, 1975.3 ± 418.6 kcal; protein, 74.6 ± 16.3 g; fat, 76.2 ± 16.3 g; carbohydrate, 234.9 ± 60.7 g; and omega-3 fatty acid, 2.1 ± 0.7 g) and EPA group (energy, 1995.4 ± 673.7 kcal; protein, 80.7 ± 38.5 g; fat, 74.0 ± 38.5 g; carbohydrate, 237.0 ± 51.8 g; omega-3 fatty acid, 1.9 ± 0.8 g) in terms of nutrition status before the intake of the supplements. The nutrition status of the participants did not change during the experimental period.

### Blood analyses

As shown in Table 1, no significant changes were observed in the PL group before and after the 8-week supplementation in terms of DGLA, AA, EPA, and DHA levels. In the EPA group, the EPA level increased after 8 weeks (*p* < 0.05). In addition, the DHA level increased after 8 weeks (*p* < 0.05). However, no significant difference was observed in the DGLA and AA levels. For comparison between groups, EPA and DHA levels were significantly higher in the EPA group (EPA; 80.3 ± 13.6 μg/ml, DHA; 104.5 ± 24.0 μg/ml) than in the PL group (EPA; 24.8 ± 14.2 μg/ml, DHA; 73.6 ± 19.6 μg/ml) after the 8-week supplementation (*p* < 0.05).

**Table 1:**
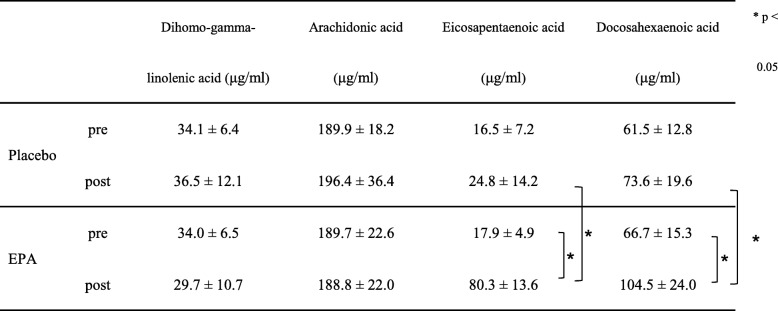
Changes of serum dihomo-gamma-linolenic acid, arachidonic acid, eicosapentaenoic acid, docosahexaenoic acid in Placebo and EPA groups at before and after 8-weeks supplementation

*p < 0.05

### Maximal voluntary isometric contraction torque

**Fig. 1:**
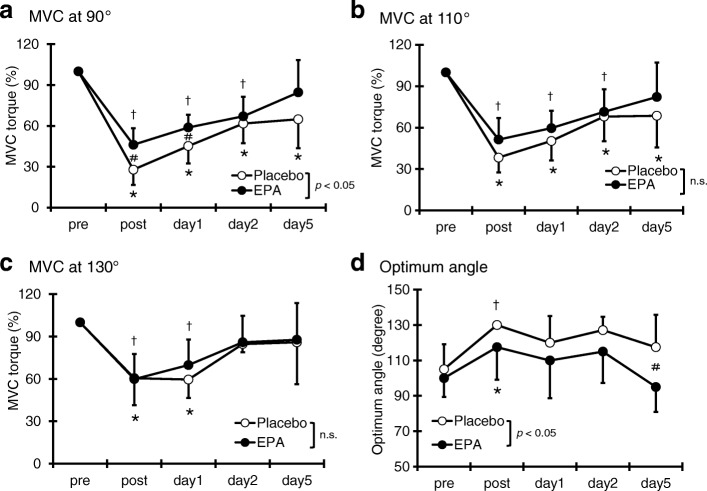
Changes (mean ± SD) of maximal voluntary isometric contraction (MVC) torque at 90° (**a**), 110° (**b**), 130° (**c**), and “optimum angle (**d**)” measured before (pre) and immediately after (post) the eccentric contractions exercise and then 1, 2 and 5 days in the placebo (PL) and EPA groups. # *p* < 0.05 for the difference between groups; * *p* < 0.05 for the difference from the pre-exercise value in the PL group, † *p* < 0.05 for the difference from pre-exercise value in the EPA group

Compared with the pre-exercise value, in the PL group, the MVC torque at 90° elbow angle significantly decreased immediately after exercise and remained until 5 days after exercise (*p* < 0.05; Fig. 1a). Moreover, in the EPA group, the MVC torque at 90° elbow angle decreased immediately after exercise and on days 1 and 2 after exercise but returned to baseline on day 5 after exercise. A significant interaction effect was found (*p* < 0.05, η^2^ = 0.14). The MVC torque at 90° elbow angle was significantly higher in the EPA group than in the PL group immediately (EPA group; 46.2 ± 12.1%, PL group; 27.9 ± 11.2%) and 1 day (EPA group; 58.8 ± 9.4%, PL group; 45.2 ± 12.8%) after exercise (*p* < 0.05). Compared with the pre-exercise value, in the PL group, the MVC torque at 110° elbow angle significantly decreased immediately after exercise and remained lower than the baseline on days 1, 2, and 5 after exercise (*p* < 0.05). In the EPA group, the MVC torque at 110° elbow angle decreased immediately and on days 1 and 2 after exercise but returned to baseline on day 5 after exercise. However, no significant difference was observed between the PL and EPA groups in terms of MVC torque at 110° elbow angle at any time points (η^2^ = 0.06; Fig. 1b). These phenomena were also observed at 130° elbow angle (η^2^ = 0.02; Fig. 1c).

### ROM of the elbow

**Fig. 2:**
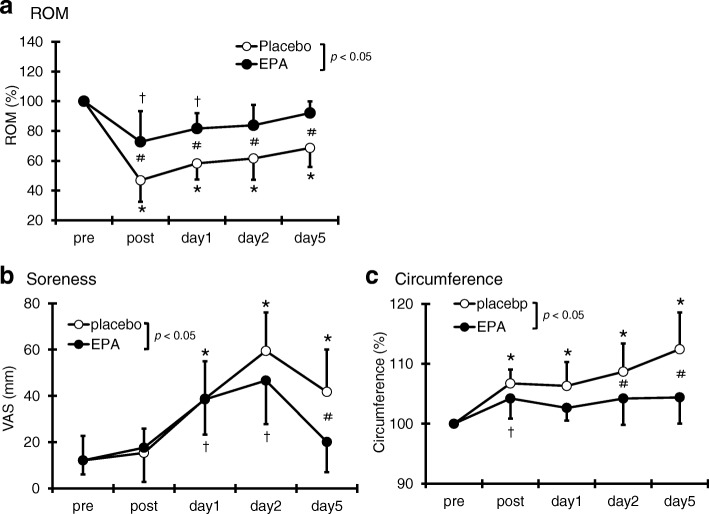
Changes (mean ± SD) of range of motion (ROM) (**a**), visual analog scale (VAS) (**b**), and circumference (**c**) measured before (pre) and immediately after (post) the eccentric contractions exercise and then 1, 2 and 5 days in the PL and EPA groups. # *p* < 0.05 for the difference between groups; * *p* < 0.05 for the difference from the pre-exercise value in the PL group, † *p* < 0.05 for the difference from pre-exercise value in the EPA group

As shown in Fig. 2a, a significant decrease in ROM was observed in the PL group immediately after exercise and remained lower than baseline on days 1, 2, and 5 after exercise. The ROM in the EPA group decreased immediately and 1 day after exercise compared with the pre-exercise value but returned to baseline on day 2 after exercise. A significant interaction effect was found (*p* < 0.05, η^2^ = 0.26). The ROM in the EPA group was significantly higher than that of the PL group immediately (EPA group; 72.8 ± 20.6%, PL group; 46.9 ± 1.4%) and after days 1 (EPA group; 81.7 ± 10.2%, PL group; 58.3 ± 10.8%), 2 (EPA group; 83.9 ± 13.7%, PL group; 61.6 ± 14.3%), and 5 (EPA group; 92.1 ± 7.8%, PL group; 68.7 ± 12.8%) after exercise (*p* < 0.05).

### Muscle soreness

Compared with the pre-exercise value, a significant level of muscle soreness was observed in the PL group using the visual analog scale on days 1, 2, and 5 after exercise (*p* < 0.05). In the EPA group, a greater muscle soreness developed on days 1 and 2 after exercise compared with the pre-exercise values. A significant interaction effect was found (*p* < 0.05, η^2^ = 0.21). The muscle soreness was significantly higher in the PL group (41.8 ± 18.3 mm) than in the EPA group (20.1 ± 13.0 mm) on day 5 after exercise (Fig. 2b).

### Upper arm circumference

The upper arm circumference of the PL group was significantly higher at all time points than the baseline value. In the EPA group, the upper arm circumference also increased immediately after exercise. However, no significant increase was observed in the upper arm circumference of the EPA group after 1 day of exercise. A significant interaction effect was found (*p* < 0.05, η^2^ = 0.27). The circumference was significantly higher in the PL group than in the EPA group on days 2 (PL group; 108.7 ± 4.7%, EPA group; 104.2 ± 4.4%) and 5 (PL group; 112.4 ± 6.1%, EPA group; 104.4 ± 4.4%) after exercise (*p* < 0.05; Fig. 2c).

### Muscle echo intensity

**Fig. 3:**
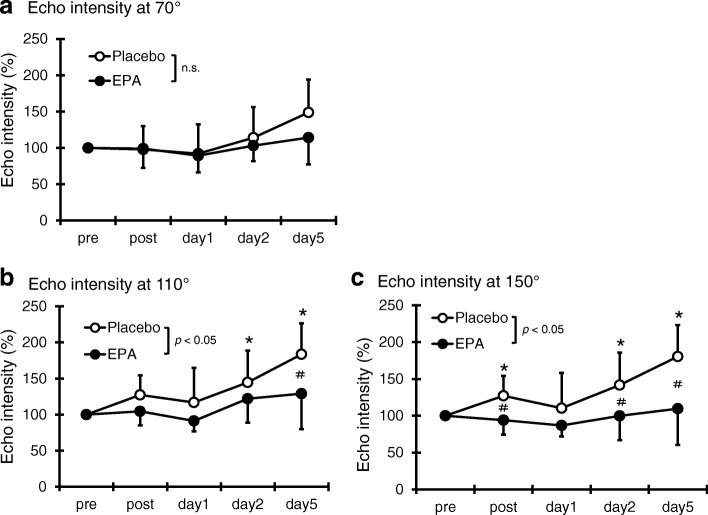
Changes (mean ± SD) of echo intensity at 70° (**a**), 110° (**b**), and 150° (**c**) measured before (pre) and immediately after (post) the eccentric contractions exercise and then 1, 2 and 5 days in the PL and EPA groups. # *p* < 0.05 for the difference between groups; * *p* < 0.05 for the difference from the pre-exercise value in the PL group, † *p* < 0.05 for the difference from pre-exercise value in the EPA group

No significant change was observed in muscle echo intensity between time and group at 70° elbow joint angle (η^2^ = 0.09, Fig. 3a). However, a significant interaction effect was found at 110°(*p* < 0.05, η^2^ = 0.19). Compared with the pre-exercise value, in the PL group, the muscle echo intensity at 110° significantly increased on days 2 and 5 after exercise. By contrast, no significant increase was observed at any time points compared with the pre-exercise value of the EPA group. The PL group (183.6 ± 42.8%) had significantly higher values than the EPA group (128.9 ± 49.3%) on day 5 after exercise (*p* < 0.05, Fig. 3b). In addition, a significant interaction effect was also found at 150°(*p* < 0.05, η^2^ = 0.30). Although the muscle echo intensity at 150° in the PL group significantly increased immediately and on days 2 and 5 after exercise, that of the EPA group did not significantly increase at any time points (Fig. 3c). The muscle echo intensity of the PL group was significantly higher than that of the EPA group immediately (PL group; 127.1 ± 25.8%, EPA group; 94.1 ± 22.8%) and on days 2 (PL group; 141.6 ± 36.2%, EPA group; 99.9 ± 28.2%) and 5 (PL group; 180.4 ± 37.2%, EPA group; 109.7 ± 26.6%) after exercise (*p* < 0.05).

### Muscle stiffness

**Fig. 4:**
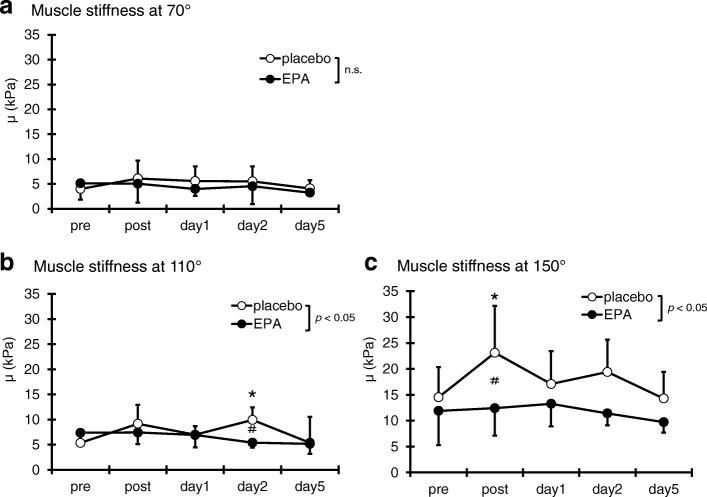
Changes (mean ± SD) of muscle stiffness at 70° (**a**), 110° (**b**), and 150° (**c**) measured before (pre) and immediately after (post) the eccentric contractions exercise and then 1, 2 and 5 days in the placebo (PL) and EPA groups. # *p* < 0.05 for the difference between groups; * *p* < 0.05 for the difference from the pre-exercise value in the PL group, † *p* < 0.05 for the difference from pre-exercise value in the EPA group

No significant difference was observed in the muscle stiffness at 70° elbow joint angle at any time points (η^2^ = 0.06, Fig. 4a). However, a significant interaction effect was found at 110°(p < 0.05, η^2^ = 0.23) and 150°(p < 0.05, η^2^ = 0.24). Compared with the pre-exercise value, in the PL group, the muscle stiffness at 110° significantly increased on day 2 after exercise (*p* < 0.05), whereas no significant change was observed in the EPA group at any time points (Fig. 4b). Compared with the value of the EPA group, the muscle stiffness at 110° in the PL group significantly increased 2 days (PL group; 9.3 ± 2.9 kPa, EPA group; 5.4 ± 1.0 kPa, p < 0.05; Fig. 4b) after the exercise, and at 150° significantly increased immediately after the exercise (PL group; 23.1 ± 9.0 kPa, EPA group; 12.4 ± 5.3 kPa, p < 0.05; Fig. 4c).

## Discussion

This study aimed to investigate the effect of EPA and DHA supplementation on the stiffness of the biceps brachii and on muscle damage after one bout of isotonic ECCs of elbow flexors. Results showed that EPA and DHA supplementations for 8 weeks inhibited the loss of muscle strength, limitation of ROM, development of muscle soreness, and increases in muscle swelling, echo intensity, and stiffness using elastographic techniques. These results supported our original hypothesis.

**Fig. 5:**
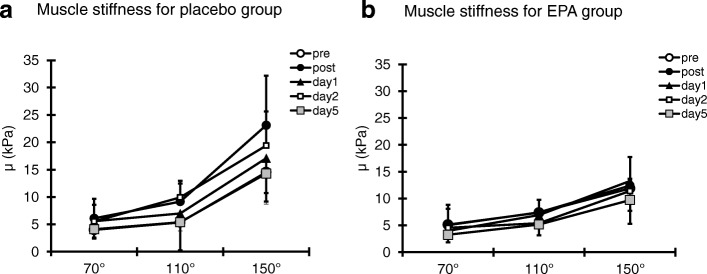
Changes (mean ± SD) of muscle stiffness for the PL group (**a**), the EPA group (**b**) at 70°, 110° and 150°

Although our previous study demonstrated that eight weeks of EPA and DHA supplementation attenuates muscle strength loss after ECCs [5], the detailed mechanism was unclear. Interestingly, this present study additionally provides evidence on the preventive effect of EPA and DHA supplementation on muscle stiffness after ECCs. Lacourpaille et al. [6] have confirmed that the early changes in muscle stiffness cannot be attributed to delayed fluid accumulation and inflammatory response after ECCs. The increase in muscle stiffness is likely associated with the loss of desmin, a cytoskeletal protein, and cell membrane damage, resulting in calcium overload [19, 20]. Hence, the early changes in muscle stiffness after ECCs reflect the rapid calcium homeostasis perturbation after the myofibrillar disruptions induced by ECCs [6, 21]. Considering these results, EPA and DHA supplementation attenuates not only delayed inflammatory response and muscle soreness but also muscle fiber disruptions. The muscle stiffness in each elbow angle is shown in Fig. 5. In the PL group, the elbow angle at 70° did not change. However, the elbow angle at 150° increased.

The reduction of MVC torque was significantly inhibited in the EPA group but not in the PL group. Our previous studies have also shown that 600-mg EPA and 260-mg DHA supplementation for 8 weeks inhibited the loss of strength after isokinetic [5] and isotonic [2] ECCs of the elbow flexors. In the present study, EPA and DHA supplementation had a preventive effect on ROM and DOMS after ECCs. These results are consistent with those of previous studies [2, 5]. Limited ROM after ECCs has been attributed to an inflammatory response in the myofibrils leading to an increase in passive stiffness and swelling [22]. DOMS is considered as micro damages in the sarcomeres that cause inflammatory responses. Our previous study has shown that 8-week EPA and DHA supplementation inhibited the increase in IL-6 level [5]. Therefore, it is suggested that the inhibitions of limited ROM and the development of DOMS could be attributed to their anti-inflammatory effects.

Previous studies have confirmed that the increase in muscle echo intensity was caused by ECCs [2, 23, 24]. Nosaka et al. [24] reported that the echo intensity of the biceps brachialis increased to approximately 30% on day 2 after ECCs and to around 60% on day 4 as a result of loading 24 maximal ECCs of elbow flexors. The increase in muscle echo intensity reflects the amount and distribution of free water or interstitial edema from extracellular matrix disruption [23]. EPA and DHA supplementation inhibited the increase in muscle echo intensity after ECCs. In addition, the increase in the upper arm circumference of the EPA group was significantly inhibited. However, our previous study reported that no significant difference between the EPA group and placebo group was observed [5]. We speculate that one of reasons for this discrepancy is caused by different exercise load, which is much greater in this study compared with previous study. Therefore, EPA and DHA may have inhibited the distribution of free water and interstitial edema by protecting the muscle membrane.

The present study had the following two limitations. First, since our protocol was one type of ECCs for untrained male subjects, further study is needed to confirm on different exercise protocols and subjects such as female, trained, etc.. Second, the sample size was relatively small in this study. It should be necessary to investigate with larger sample size.

## Conclusion

In summary, EPA and DHA supplementation inhibited muscle stiffness. In addition, EPA and DHA supplementation for 8 weeks inhibited the loss of muscle strength, limitation in ROM, development of DOMS, and increases in muscle swelling and echo intensity. These results may be significant in identifying the mechanism associated with the preventive effect of EPA and DHA supplementation on muscle damage.

## Abbreviations

AA: Arachidonic acid
BMI: Body mass index
DGLA: Dihomo-gamma-linolenic acid
DHA: Docosahexaenoic acid
DOMS: Delayed onset muscle soreness
ECCs: Eccentric contractions
EPA: Eicosapentaenoic acid
IL-6: Interleukin-6
MVC: Maximal voluntary contraction
PL: Placebo
ROM: Range of motion

## References

[1] Helge JW, Therkildsen KJ, Jorgensen TB (2001). Eccentric contractions affect muscle membrane phospholipid fatty acid composition in rats. Exp Physiol.

[2] Ochi E, Tsuchiya Y, Yanagimoto K (2017). Effect of eicosapentaenoic acids-rich fish oil supplementation on motor nerve function after eccentric contractions. J Int Soc Sports Nutr.

[3] Tartibian B, Maleki BH, Abbasi A (2009). The effects of ingestion of omega-3 fatty acids on perceived pain and external symptoms of delayed onset muscle soreness in untrained men. Clin J Sport Med.

[4] Tartibian B, Maleki BH, Abbasi A (2011). Omega-3 fatty acids supplementation attenuates inflammatory markers after eccentric exercise in untrained men. Clin J Sport Med.

[5] Tsuchiya Y, Yanagimoto K, Nakazato K (2016). Eicosapentaenoic and docosahexaenoic acids-rich fish oil supplementation attenuates strength loss and limited joint range of motion after eccentric contractions: a randomized, double-blind, placebo-controlled, parallel-group trial. Eur J Appl Physiol.

[6] Lacourpaille L, Nordez A, Hug F (2014). Time-course effect of exercise-induced muscle damage on localized muscle mechanical properties assessed using elastography. Acta Physiol (Oxf).

[7] Green MA, Sinkus R, Gandevia SC (2012). Measuring changes in muscle stiffness after eccentric exercise using elastography. NMR Biomed.

[8] Gennisson JL, Deffieux T, Mace E (2010). Viscoelastic and anisotropic mechanical properties of in vivo muscle tissue assessed by supersonic shear imaging. Ultrasound Med Biol.

[9] Shinohara M, Sabra K, Gennisson JL (2010). Real-time visualization of muscle stiffness distribution with ultrasound shear wave imaging during muscle contraction. Muscle Nerve.

[10] Debernard L, Robert L, Charleux F (2011). Characterization of muscle architecture in children and adults using magnetic resonance elastography and ultrasound techniques. J Biomech.

[11] Dresner MA, Rose GH, Rossman PJ (2001). Magnetic resonance elastography of skeletal muscle. J Magn Reson Imaging.

[12] Koo TK, Guo JY, Cohen JH (2014). Quantifying the passive stretching response of human tibialis anterior muscle using shear wave elastography. Clin Biomech (Bristol, Avon).

[13] Maisetti O, Hug F, Bouillard K (2012). Characterization of passive elastic properties of the human medial gastrocnemius muscle belly using supersonic shear imaging. J Biomech.

[14] Administration (2000). UFaD: letter regarding dietary supplement health claim for omega-3 fatty acids and Coro- nary heart disease.

[15] Sasaki K, Sasaki T, Ishii N (2011). Acceleration and force reveal different mechanisms of electromechanical delay. Med Sci Sports Exerc.

[16] Tsuchiya Y, Kikuchi N, Shirato M (2015). Differences of activation pattern and damage in elbow flexor muscle after isokinetic eccentric contractions. Isokinet Exerc Sci.

[17] Ochi Eisuke, Tsuchiya Yosuke, Nosaka Kazunori (2016). Differences in post-exercise T2 relaxation time changes between eccentric and concentric contractions of the elbow flexors. European Journal of Applied Physiology.

[18] Akagi R, Tanaka J, Shikiba T (2015). Muscle hardness of the triceps brachii before and after a resistance exercise session: a shear wave ultrasound elastography study. Acta Radiol.

[19] Lieber RL, Thornell LE, Friden J (1996). Muscle cytoskeletal disruption occurs within the first 15 min of cyclic eccentric contraction. J Appl Physiol (1985).

[20] Balnave CD, Allen DG (1996). The effect of muscle length on intracellular calcium and force in single fibres from mouse skeletal muscle. J Physiol.

[21] Lacourpaille L, Nordez A, Hug F (2017). Early detection of exercise-induced muscle damage using elastography. Eur J Appl Physiol.

[22] Chleboun GS, Howell JN, Conatser RR (1998). Relationship between muscle swelling and stiffness after eccentric exercise. Med Sci Sports Exerc.

[23] Nosaka K, Clarkson PM (1996). Changes in indicators of inflammation after eccentric exercise of the elbow flexors. Med Sci Sports Exerc.

[24] Nosaka K, Newton M, Sacco P (2005). Partial protection against muscle damage by eccentric actions at short muscle lengths. Med Sci Sports Exerc.

